# Prevalence, molecular epidemiology, and antimicrobial resistance of methicillin-resistant *Staphylococcus aureus* from swine in southern Italy

**DOI:** 10.1186/s12866-019-1422-x

**Published:** 2019-02-26

**Authors:** Mattia Pirolo, Angela Gioffrè, Daniela Visaggio, Monica Gherardi, Grazia Pavia, Pasquale Samele, Lucia Ciambrone, Rossella Di Natale, Giovanna Spatari, Francesco Casalinuovo, Paolo Visca

**Affiliations:** 1 Department of Science, Roma Tre University, Viale G. Marconi 446, 00146 Rome, Italy; 2 Department of Medicine, Epidemiology, Workplace and Environmental Hygiene, Lamezia Terme Research Centre, INAIL – National Institute for Insurance against Accidents at Work, Lamezia Terme, Italy; 3 Department of Medicine, Epidemiology, Workplace and Environmental Hygiene , Monte Porzio Catone Research Centre, INAIL – National Institute for Insurance against Accidents at Work, Rome, Italy; 4 Istituto Zooprofilattico Sperimentale del Mezzogiorno, Catanzaro, Italy; 5 Department of Biomedical Sciences, Dental, Morphological and Functional Investigations, University of Messina, Messina, Italy

**Keywords:** LA-MRSA, Pigs, Genotyping, Antimicrobial susceptibility, Southern Italy

## Abstract

**Background:**

Colonization by livestock-associated MRSA (LA-MRSA) has increasingly been reported in the swine population worldwide. The aim of this study was to assess the prevalence of MRSA nasal carriage in healthy pigs, including the black (*Calabrese*) breed, from farms in the Calabria Region (Southern Italy). Between January and March 2018, a total of 475 healthy pigs reared in 32 farms were sampled by nasal swabbing. MRSA isolates were characterized by *spa*, MLST and SCC*mec* typing, and susceptibility testing to 17 antimicrobials.

**Results:**

22 of 32 (66.8%) pig farms resulted positive for MRSA. The prevalence of MRSA was 46.1% (219 MRSA culture-positive out of 475 samples). MRSA colonization was significantly higher in intensive farms and in pigs with a recent or ongoing antimicrobial treatment. All 219 MRSA isolates were assigned to ST398. The most common *spa* types were t011 (37.0%), t034 (22.4%) and t899 (15.1%). A novel *spa* type (t18290) was detected in one isolate. An insertion of IS*256* in the ST398-specific A07 fragment of the SAPIG2195 gene was detected in 10 out of 81 t011 isolates. Nearly all isolates carried the SCC*mec* type V element, except 11 isolates that carried the SCC*mec* type IVc. None of the isolates was positive for the Panton-Valentine leukocidin. All isolates were resistant to tetracycline. High resistance rates were also found for clindamycin (93.1%), trimethoprim/sulfamethoxazole (68.4%), fluoroquinolones (47.9–65.3%) and erythromycin (46.1%). None of the isolates was resistant to vancomycin and fusidic acid. Overall, a multidrug resistant phenotype was observed in 88.6% of isolates.

**Conclusions:**

We report a high prevalence of MRSA among healthy swine in Southern Italy farms, with higher isolation frequency associated with intensive farming. The epidemiological types identified in our study reflect those reported in other European countries. Our findings underscore the importance of monitoring the evolution of LA-MRSA in pig farms in order to implement control measures and reduce the risk of spread in the animal population.

**Electronic supplementary material:**

The online version of this article (10.1186/s12866-019-1422-x) contains supplementary material, which is available to authorized users.

## Background

Antimicrobial resistance is a looming public health crisis that threatens the effective prevention and treatment of infectious diseases. The development of antimicrobial resistance is accelerated by the misuse and overuse of antibiotics in human and veterinary medicine, animal farming and in agricultural settings [1]. In the modern animal husbandry antimicrobials are employed in large quantities to treat and prevent bacterial diseases [2]. The World Health Organization has urged a ban on growth-promoting antibiotics in fatten farm animals [3], a practice already banned in European Union (EU) and United States [4]. Despite these precautions, antimicrobial resistance among bacterial pathogens in the animal husbandry is progressively increasing, contributing to the spread of multi-drug resistant (MDR) microorganism in the community [5].

Methicillin-resistant *Staphylococcus aureus* (MRSA) has for long time been considered a prototypic nosocomial pathogen, showing highest prevalence in healthcare-associated infections (HA-MRSA) [6]. This view has changed over the last decades, since MRSA has become increasingly frequent in community acquired infections (CA-MRSA) in healthy people [7]. Furthermore, the high prevalence of MRSA in both pets and livestock highlights animals as a worrisome reservoir of this pathogen [8, 9]. Livestock-associated MRSA (LA-MRSA) is considered a serious concern for the risks of zoonotic transmission, not only to people with occupational livestock exposure [10, 11], but also to the community through the food chain [12]. The foremost common LA-MRSA worldwide is the sequence type (ST) 398 belonging to clonal complex (CC) 398 [13]. Although LA-MRSA ST398 has been isolated from different livestock animals (i.e. veal calves, poultry, horses) [14–16], the main reservoir for this clone are pigs [13]. After the first isolation from pigs in France [17], ST398 was increasingly detected throughout Europe, accounting for 92.5% of the MRSA isolates from breeding or production holdings of 17 EU Member States [18]. As a foreseeable consequence, a recent survey by the European Centre for Disease Prevention and Control (ECDC) reports an increased prevalence of LA-MRSA in humans between 2007 and 2013 (from 1.7 to 3.9%; ref. [19]. Therefore, in line with the “One World, One Health” principles [20], an integrated multi-sectorial surveillance including both healthcare and veterinary sources, to systematically map potential reservoirs and epidemiological trajectories of MRSA, has become mandatory [19].

In Italy, national surveillance data on MRSA are available only for nosocomial infections, in which MRSA account for 33.9% of *S. aureus* isolates from invasive infections in 2017 [21]. In contrast, systematic veterinary surveillance of LA-MRSA has not yet been established. However, some studies reported an extremely high prevalence (34.9–38.1%) of LA-MRSA from pig holdings in Italy [18, 22]. These percentages reflect those seen in other EU countries with high density of swine farming, such as Germany (50–52%) [23, 24], Spain (46%) [25] and Belgium (44%) [26]. An estimated MRSA prevalence of 37.6% was recently reported in slaughtered pigs of two industrial abattoirs in Southern Italy [27].

Given the serious threat of zoonotic MRSA transmission and the high isolation rate of LA-MRSA from intensive pig farms in Italy, the aims of the present study were: (i) to assess the prevalence of MRSA among asymptomatic swine, including the autochthonous black (*Calabrese*) pig breed [28, 29], from farms located in the Calabria region (Southern Italy); (ii) to investigate differences in MRSA carriage between intensive and non-intensive farming; (iii) to determine the clonal profiles of pig-associated MRSA isolates; (iv) to investigate the antimicrobial resistance patterns and the staphylococcal chromosomal cassette *mec* (SCC*mec*) type of MRSA isolates.

## Results

### Prevalence of pig-associated MRSA

The nasal carriage of MRSA in healthy swine reared in 32 farms with different type of breeding (25 intensive; 7 non-intensive) in all provinces of Calabria region (Additional file 1: Figure S1) was estimated. The characteristics of selected farms are summarized in Table 1.

**Table 1:** Characteristics of pig farms

Farm ID	Province	Breeding type	No. of farmed pigs	No. of sampled pigs (including black pigs)	No. of antibiotic-treated among sampled pigs^a^
01CZ	Catanzaro	Intensive	275	12 (0)	3
02CZ	Catanzaro	Intensive	380	10 (1)	6
03CZ	Catanzaro	Intensive	300	10 (0)	8
04CZ	Catanzaro	Intensive	20	14 (0)	0
05CS	Cosenza	Intensive	3967	29 (0)	0
06CS	Cosenza	Non-intensive	60	6 (6)	0
07KR	Crotone	Intensive	2309	19 (2)	2
08CZ	Catanzaro	Intensive	1010	18 (0)	0
09CZ	Catanzaro	Intensive	10	8 (0)	1
10RC	Reggio Calabria	Intensive	341	18 (0)	0
11RC	Reggio Calabria	Intensive	158	13 (0)	13
12RC	Reggio Calabria	Non-intensive	99	10 (10)	0
13CZ	Catanzaro	Intensive	50	15 (0)	0
14CZ	Catanzaro	Intensive	20	12 (0)	0
15CZ	Catanzaro	Intensive	120	15 (0)	0
16CS	Cosenza	Non-intensive	170	17 (17)	0
17CS	Cosenza	Non-intensive	1207	11 (11)	0
18CS	Cosenza	Intensive	600	10 (0)	0
19RC	Reggio Calabria	Intensive	124	20 (0)	0
20RC	Reggio Calabria	Non-intensive	150	15 (15)	0
21RC	Reggio Calabria	Intensive	330	16 (0)	0
22CZ	Catanzaro	Intensive	850	30 (0)	0
23CZ	Catanzaro	Intensive	400	20 (0)	20
24KR	Crotone	Intensive	144	20 (0)	0
25KR	Crotone	Intensive	300	14 (0)	0
26VV	Vibo Valentia	Intensive	132	23 (3)	0
27VV	Vibo Valentia	Non-intensive	24	10 (10)	0
28VV	Vibo Valentia	Intensive	10	9 (1)	0
29RC	Reggio Calabria	Intensive	765	20 (0)	7
30RC	Reggio Calabria	Non-intensive	32	2 (2)	0
31RC	Reggio Calabria	Intensive	1101	20 (0)	0
32RC	Reggio Calabria	Intensive	50	9 (0)	0

^a^Ongoing or suspended in the last twenty days

From January to March 2018, a total of 475 nasal swabs were obtained from pigs (2 to 29 sampled animals per farm). Sampling and processing procedures for *S. aureus* detection and MRSA isolation from nasal swabs are outlined in Additional file 2: Figure S2 (see also Materials and Methods for details).

All farms resulted positive for the presence of *S. aureus*, with an overall prevalence of 82.1% (95% CI: 81.8–82.4%; Table 2); among these, more than half (22 out of 32 farms) were also positive for the presence of MRSA, with a prevalence of 46.1% (95% CI: 45.9–46.3%). A total of 219 non-duplicate MRSA isolates were obtained (Table 2).

**Table 2:** Prevalence of *S. aureus* according to breeding type, breed and antimicrobial treatment

Variable	Category	No. of farms	No. of sampled animals	*S. aureus-*positive		MRSA-positive	
				No. (%)	*P*-value	No. (%)	*P*-value
Breeding type	Intensive	25	405	350 (86.4)	NS	218 (53.8)	> 0.001
	Non-intensive	7	70	40 (57.1)		1 (1.4)	
	Total	32	475	390 (82.1)		219 (46.1)	
Breed	Black pig	11^a^	77	47 (61.0)	NS	7 (9.1)	> 0.001
	Other breeds	25	398	343 (86.2)		212 (53.3)	
	Total	32	475	390 (82.1)		219 (46.1)	
Antimicrobial treatment	Treated^b^	–	60	57 (95.0)	NS	45 (75.0)	0.010
	Untreated	–	415	333 (80.2)		174 (41.9)	
	Total	–	475	390 (82.1)		219 (46.1)	

^a^A minority of black pigs were occasionally reared also in 4 intensive farms

^b^Ongoing or suspended in the last twenty days

NS, Not significant

Regarding *S. aureus* colonization, no significant differences in detection frequency were observed between intensive and non-intensive farms, pig breed (black vs. other breeds), and antimicrobial treatment (untreated vs. treated) (Table 2). Conversely, MRSA isolation was significantly higher among intensive than non-intensive farms (53.8% versus 1.4%, *p* > 0.001; Table 2). As a consequence, the black pig, which was almost exclusive reared in non-intensive farms (Table 1), showed significantly lower colonization by MRSA than other breeds (9.1% versus 53.3%, *p* > 0.001; Table 2). Finally, the MRSA isolation rate was significantly higher in pigs with an ongoing or recent antibiotic treatment (suspended in the last twenty days) than in untreated animals (75% versus 41.9%, *p* = 0.01; Table 2).

### Clonal profiles of pig-associated MRSA isolates

**Fig. 1:**
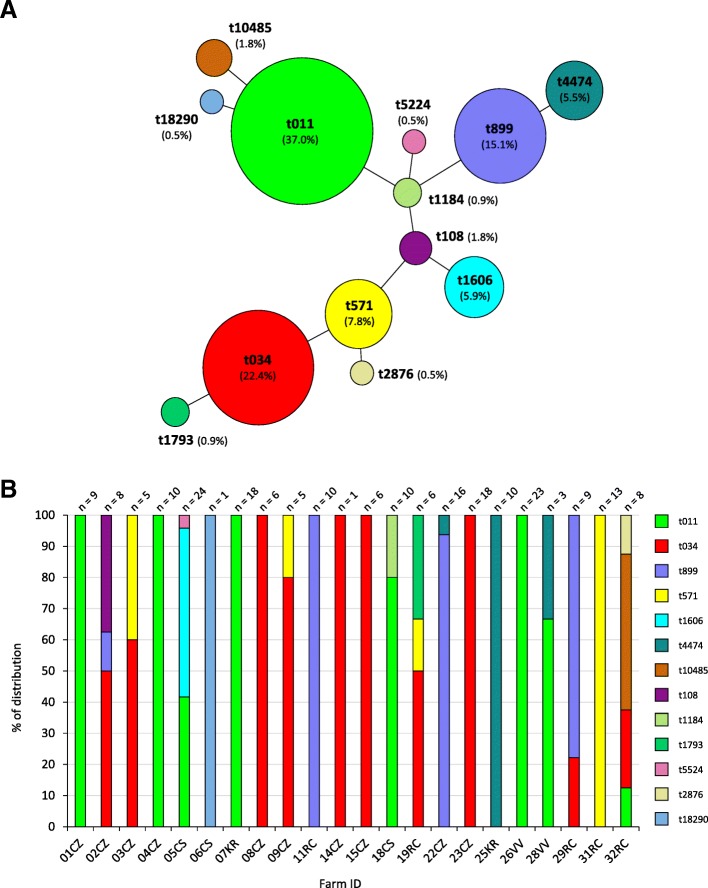
Frequency and distribution of *spa* types for sampled farms. **a** Minimum spanning tree based on *spa* types for all MRSA isolates (*n* = 219). Each node represents a different *spa* type. The diameter of node is proportional to the number of the isolates belonging to the *spa* type. **b** Distribution of *spa* types among 32 screened farms

To determine the clonal profile of the pig-associated MRSA, *spa* typing, Multi Locus Sequence Typing (MLST) and SCC*mec* typing were performed. Thirteen different *spa* types were identified, and a minimum spanning tree was generated showing the type frequency and the genetic distance between types (Fig. 1a). The majority of isolates belonged to *spa* type t011 (81/219, 37.0%), t034 (49/219, 22.4%) and t899 (33/219, 15.1%). One new *spa* type, t18290, was detected in a black pig from a non-intensive farm (ID 06CS) (Fig. 1b). This new *spa* type is closely related to t011 since it differs by a single-nucleotide substitution in the third repeat (repeat 783 in t18290, instead of 02 in t011) (Additional file 3: Table S1). In 12 out of 22 (54.5%) MRSA-positive farms, a single *spa* type was detected, whereas in the remaining 10 farms (45.5%) two or more *spa* types were detected (Fig. 1b).

During our survey, a trade of pigs was documented between two farms, ID 07KR (seller) and 18CS (purchaser). Intriguingly, MRSA isolates from these two farms belonged to the t011 (18/18 in farm 07KR and 8/10 in 18CS; Fig. 1b).

**Fig. 2:**
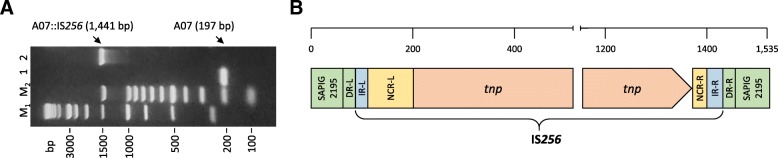
IS*256* insertion in the A07 fragment of the SAPIG2195 coding region. **a** ST398-specific PCR using primers A07f/A07r [30] of different t011 MRSA isolates from farm ID 05CS. Lanes 1 and 2, A07 (197 bp) and A07::IS*256* (1535 bp), respectively. Lanes M_1_ and M_2_, 1 kb and 100 bp molecular size markers (Promega) respectively. **b** Schematic of the IS*256* element inserted in the A07 fragment of SAPIG2195 [31]. The transposase gene (*tnp*) is flanked by non-coding regions (NCR-L and NCR–R) that harbor imperfect inverted repeats (IR-L and IR-R). The two octanucleotide direct repeats (DR-L and DR-R) flank the IS*256*

The majority of *spa* types identified in this study have previously been associated to ST398. In order to verify this association, all isolates were screened by ST398-specific PCR [30]. This PCR generates an amplicon of 197 bp, corresponding to the fragment A07 of the SAPIG2195 gene (Gene ID: 12322222) [31]. As expected, an amplicon of 197 bp was obtained for 209/219 MRSA isolates, suggesting that they belong to ST398. Of note, an amplicon of 1535 bp was detected in 10/219 MRSA, all isolated from farm ID 05CS and belonging to t011 (Fig. 2a). DNA sequence analysis of the 1535 bp amplicon revealed the presence of a 1329 bp insertion in SAPIG2195. The inserted DNA displayed 99% sequence identity with a genomic region of *S. aureus* WCH-SK2 (genome ID: CP031537; nucleotides 1,708,473–1,709,814) corresponding to IS*256*, including the 5′ and 3′ octanucleotide direct repeats (DR-L and DR-R) originated from the transposition event [32, 33]. The IS*256* consists of a transposase gene (*tnp*) flanked by non-coding regions (NCR-L and NCR–R) that harbor 26 bp imperfect inverted repeats (IR-L and IR-R) (Fig. 2b and Additional file 4: Figure S3).

To confirm that all the MRSA belonged to ST398, one MRSA isolate for each *spa* type was analysed by MLST, including one t011 strain that harboured the IS*256* element in the SAPIG2195 gene. As expected, all MRSA isolates belonged to ST398, showing the allelic profile 3–35–19-2-20-26-39.

The vast majority (95.0%) of MRSA isolates carried the SCC*mec* type V element, while few isolates, belonging to t899, carried SCC*mec* type IVc (5.0%) (Additional file 3: Table S1). Finally, all the isolates were screened for the presence of the Panton-Valentine leucocidin (PVL) genes (*lukS*/*lukF*). Of note, none of the MRSA isolates harboured the PVL genes.

### Antimicrobial susceptibility

Antimicrobial susceptibility testing results on the 219 MRSA isolates are shown in Table 3. All isolates showed resistance to penicillin (PEN), oxacillin (OXA) and tetracycline (TET) and the majority of them was also resistant to clindamycin (CLI; 93.1%), trimethoprim-sulfamethoxazole (SXT; 68.4%), ampicillin/sulbactam (AMS; 66.2%) and enrofloxacin (ENR; 65.3%). Intriguingly, 48.9% of the MRSA isolates showed the lincosamide-resistant/macrolide-susceptible phenotype; more than half (52.5%) of CLI-resistant isolates were susceptible to erythromycin (ERY). All the MRSA were susceptible to vancomycin (VAN) and fusidic acid (FUS) (Table 3). Nearly 90% of the MRSA isolates (213/219) were MDR, resulting resistant to at least three non β-lactams antimicrobial classes.

**Table 3:** Resistance to individual antimicrobials in 219 MRSA isolates from pigs

Antimicrobial target	Antimicrobial class	Antimicrobial^a^	No. of non-susceptible (R + I) isolates (%)^b^
Peptidoglycan synthesis	β-lactams	*PEN*	219 (100)
		*OXA*	219 (100)
		*AMS*	145 (66.2)
	Carbapenems	*IMP*	22 (10.0)
	Glycopeptides	*VAN*	0 (0)
DNA synthesis	Fluoroquinolones	*ENR*	143 (65.3)
		*MAR*	105 (47.9)
Protein synthesis	Aminoglycosides	*GEN*	43 (19.6)
		*KAN*	47 (21.5)
	Macrolides	*ERY*	101 (46.1)
	Lincosamides	*CLI*	204 (93.1)
	Tetracyclines	*TET*	219 (100)
	Fucidanes	*FUS*	0 (0)
	Phenicols	*CHL*	16 (7.3)
	Ansamycins	*RIF*	1 (0.5)
Others	Nitrofuranes	*NIT*	10 (4.6)
	Folate pathways inhibitors	*SXT*	150 (68.4)

^a^Acronyms: PEN, penicillin; OXA, oxacillin; AMS, ampicillin/sulbactam; IMP, imipenem; VAN; vancomicin; ENR, enrofloxacin; MAR, marbofloxacin; GEN, gentamycin; KAN, kanamycin; ERY, erythromycin; CLI, clindamycin; TET, tetracycline; FUS, fusidic acid; CHL, chloramphenicol; RIF, rifampicin; NIT, nitrofurantoin; SXT, trimethoprim-sulfamethoxazole

^b^Isolates showing resistance (R) and intermediate susceptibility (I) were classified as non-susceptible

**Fig. 3:**
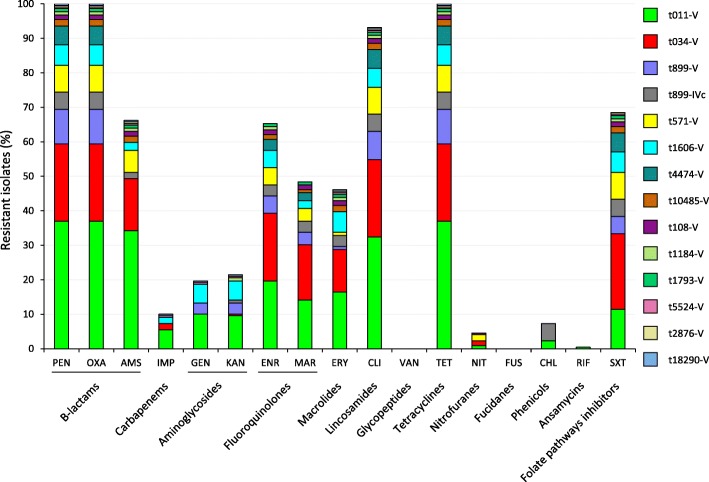
Distribution of epidemiological types (combined *spa* and SCC*mec* type) according to the resistance to individual antimicrobial compounds

Overall, 77 resistance profiles were detected (Additional file 5: Table S2), with an antibiotype diversity of 0.54 (Additional file 6: Table S3). As a consequence, no correlation could be determined between the antibiotic resistance profile and *spa* or *SCCmec* type (Additional file 5: Table S2). The most frequent antibiotype, determined for 22/219 (10.1%) isolates, was OXA-PEN-AMS-ENR-marbofloxacin (MAR)-ERY-CLI-TET-SXT (Additional file 5: Table S2). The *spa* and SCC*mec* type distribution according to individual antimicrobial resistances is illustrated in Fig. 3 and details are provided in Additional file 6: Table S3.

## Discussion

Following the European recommendations and given the high rate of MRSA isolation from livestock animals in EU, systematic monitoring and epidemiological characterization of circulating MRSA strains have become fundamental components of health and safety plans in many EU countries [34]. Our study represents the first systematic survey of MRSA carriage in swine within a defined region of Southern Italy. Calabria was chosen since it is a primary producer of several autochthonous pork products (e.g. *capocollo, pancetta, sausage, nduja*), highly appreciated by the national and international market, and included in the list of Italian Protected Designation of Origin (PDO), as defined in the Council Regulation CE 510/2006. After sampling 475 pigs in 32 farms (Additional file 1: Figure S1; Table 1), the observed MRSA prevalence was 46.1%, thus comparable with that reported for other Italian regions [27] and EU countries [23–26]. However, the MRSA prevalence was much higher in farms with an intensive type of breeding compared to non-intensive type (53.8% versus 1.4%, *p* > 0.001) (Table 2). This finding is in accordance with previous reports demonstrating a strict correlation between the frequency of MRSA isolation and the crowded environment in the holdings [23, 25, 35–37], as opposed to organic farms (non-intensive holdings) in which the prevalence of LA-MRSA much lower [38, 39]. Accordingly, in our survey one out of 70 pigs reared in organic farms (1.4%) was MRSA-positive (Table 2). This further corroborates the notion that the herd’s management plays a key role in the containment of MRSA spreading. Moreover, the black (*Calabrese*) pig breed, which is one of the six Italian autochthonous pig breeds [28, 29], showed much lower colonization rates compared with other breeds (9.1% versus 53.3%, *p* > 0.001) (Table 2). This difference can be attributed to the fact that the black (*Calabrese*) pig is predominantly reared in non-intensive holdings, as opposed to the other breeds.

In EU countries, LA-MRSA isolates most often belong to ST (CC)398 [18], and are distributed in a large variety of *spa* types [19, 40]. Accordingly, all 13 *spa* types identified in this study, including the new *spa* type t18290, belonged to ST398. Interestingly, in 54.5% of the farms, pigs were colonized with a single *spa* type, whereas in the remaining 45.4% the co-occurrence of two to four *spa* types was observed (Fig. 1b). Inter-farm trade of MRSA-positive swine can partly explain the occurrence of different *spa* types in the same holding. In fact, trade of animals between two holdings was documented in this study, and sampled animals from both farms were colonized with MRSA belonging to the same *spa* type (Fig. 1b).

In line with previous Italian studies [22, 27], the predominant *spa* types were t011, t034 and t899, accounting for 74.5% of all isolates (Fig. 1a). Interestingly, 10 isolates reared in the same farm (ID 05CS) and belonging to t011 were all characterized by the insertion of IS*256* in the A07 fragment of the SAPIG2195 gene (Fig. 2). The IS*256* is an insertion sequence that confers a strong genomic plasticity to MRSA [41]. Since IS*256* is present in multiple copies in the staphylococcal genome [32], the dimension of the polymorphic inter-IS*256* sequences has previously been used as a typing tool for MRSA [42]. This feature provides compelling evidence of the close genetic relatedness of this cluster of t011 isolates.

Regarding SCC*mec* element in our isolates, the most prevalent was type V (208 isolates, 95%), which is frequently present in pig-associated ST398 MRSA [23, 40, 43]. The remaining 11 isolates (5%), all belonging to t899, harboured the SCC*mec* type IVc (Additional file 3: Table S1), which is more common in CA-MRSA, as opposed to type IVa that prevails in LA-MRSA [44–46]. Moreover, the PVL genes were not detected in our collection of pig-associated MRSA, consistent with previous observations [22, 37, 47] and with the notion that PVL is a prevalent trait of CA-MRSA [48].

A typical feature of LA-MRSA is the ability to resist to several antimicrobials [26, 47, 49]. This has been ascribed to overuse and misuse of antibiotics in the animal husbandry, which drove the selection and evolution of resistance. The European Medicines Agency (EMA) and the European Surveillance of Veterinary Antimicrobial Consumption (ESVAC) reported a massive reduction of sales of veterinary antimicrobials in 24 out of 30 EU countries, including Italy (30% reduction), between 2010 and 2016 [50]. Despite this policy, our survey highlights an overall high rate of antimicrobial resistance in pig-associated MRSA (Table 3), resulting 88.6% of isolates resistant to three or more classes of non β-lactams antimicrobials. Notably, MRSA prevalence was significantly higher in pigs with a recent or ongoing antimicrobial treatment, compared with untreated animals (75% versus 41.9%, *p* = 0.01; Table 2), as also observed in other surveys [23, 35].

Seventy-seven different antimicrobial susceptibility profiles were identified among 219 LA-MRSA isolates (antibiotype diversity 0.54; Additional file 5: Table S2 and Additional file 6: Table S3), denoting extensive variability of antimicrobial resistance combinations within individual *spa* types. Tetracycline resistance is a hallmark of ST398 LA-MRSA from swine in Europe [22–25, 27, 37, 49], being the consequence of extensive usage of chlor- and oxy-tetracycline in pig farming [51]. This holds true also for lincosamides, macrolides and fluoroquinolones [52, 53]. Indeed, all our isolates were invariably resistant to TET, and showed high to medium frequency of resistance to CLI (93.1%), ERY (46.1%), ENR (65.3%) and MAR (47.9%) (Table 3). Of note, the atypical lincosamide-resistant/macrolide-susceptible phenotype was observed for nearly half of the isolates. This resistance profile has increasingly been reported among ST398 MRSA from swine [54], and can be attributed to the spread of mobile genetic elements carrying the pleuromutilin-lincosamide-streptogramin A resistance genes (*vga* alleles) among swine-associated ST398 MRSA [55, 56]. A lower rate of gentamicin (GEN) and kanamycin (KAN) resistance (19.6 and 21.5%, respectively) (Table 3) was observed, compared with previous reports from Italy (30%; ref. [22] and other EU countries (35–45%; refs. [23, 26, 40, 57]. Resistance to chloramphenicol (CHL), rifampicin (RIF), and FUS was infrequent or absent (7.3, 0.5 and 0%, respectively), as in previous surveys [24, 49, 57–59].

SXT deserves a special comment. In the last years, an increase of SXT resistance in pig-associated MRSA has been documented, with percentages of resistance varying from 30 to 44% [23, 37, 59]. Here, we report an alarmingly high rate of SXT resistance (68.4%), which in the future could further increase as a consequence of horizontal transmissibility of the *dfrK* gene, encoding for trimethoprim-resistance [60]. Such high frequency of SXT resistance is probably due the selective pressure imposed by long-term exposure of animals to this drug, given that SXT is used as a metaphylactic (individual animal) or prophylactic (whole herd) preventive agent in intensive pig farming [61]. In our setting, however, such exposure cannot be proven, due to poor or incomplete information on antibiotic management available from farmers.

Remarkably, swine-associated MRSA from our study were invariably susceptible to VAN (Table 3), which remains the drug of choice for treatment of MRSA infections in humans [62].

## Conclusions

The present study highlights the high prevalence of LA-MRSA ST398 among healthy pigs in intensive holdings in Southern Italy, as opposed to the low prevalence in non-intensive holdings. Circulating ST and *spa* types largely reflect the clonal distribution of LA-MRSA in the Italian and European pig farming industry. Susceptibility testing revealed extensive resistance to different classes of antimicrobials, especially those commonly used in pig husbandry. A worrisome increase in SXT resistance was observed, which deserves future attention. The high prevalence of ST398 LA-MRSA in intensive animal husbandry, together with its growing antimicrobial resistance, underscores the importance of monitoring the evolution of LA-MRSA in pig farms in order to implement control measures and reduce the risk of spread in the animal population.

## Methods

### Study design and samples collection

Between January and March 2018, a cross-sectional study was carried out in 32 swine herds located in all the five provinces of the Calabria region (15,082 km^2^): Catanzaro (CZ; 11 farms), Reggio Calabria (RC; 10 farms), Cosenza (CS; 5 farms), Vibo Valentia (VV; 3 farms), Crotone (KR; 3 farms) (Additional file 1: Figure S1). Farms were selected by both geographic distribution and convenience, mainly based on the disposition of farmers to participate to the survey. The farms belonged to intensive (25/32) and non-intensive (7/32) types of breeding. Intensive-type farms were considered those in which animals were in crowded conditions. In these farms, swine were mainly represented by hybrids, deriving from crossings of different breeds (Large White, Durok, Danish and Polish). Swine holdings with non-intensive breeding systems were those in which animals were not confined between fences. In these farms, the autochthonous (*Calabrese*) black pig was the only reared breed.

Swab samples were collected from the nose of 475 animals, corresponding to 0.7 to 90% of the total swine livestock in each farm, depending on herd size (Table 1). Plastic swabs were pre-moistened in a sterile 0.9% NaCl solution in order to increase the isolation rate of *S. aureus*, as previously documented [63]. Each swab was placed in a 15-ml tube containing 5 ml of Mueller Hinton Broth (MHB) (Becton Dickinson) supplemented with 6.5% (w/vol) sodium chloride (NaCl). Tubes were incubated for 24 h at 37 °C.

### Detection and isolation of *S. aureus* and MRSA

All samples were processed according to a previously described protocol [64], with modifications (Additional file 2: Figure S2). Briefly, after 24-h swab incubation in MHB + 6.5% NaCl at 37 °C, 0.5-ml aliquots were transferred to 4.5 ml of Phenol-Red Mannitol Broth (PRMB) (Becton Dickinson) and 4.5 ml PRMB supplemented with 4 μg/ml of oxacillin (PRMB + OX). The two tubes, obtained from the same initial sample, were incubated for 24–48 h at 37 °C. If red-to-yellow colour change was observed in both samples (PRMB and PRMB + OX), 10-μl from the culture with oxacillin (PRMB + OX) were streaked on selective plates for MRSA (Brilliance MRSA 2 agar, Oxoid). Suspected MRSA colonies (blue coloured) were further streaked in Muller Hinton Agar (MHA) (Becton Dickinson) supplemented with 4 μg/ml of oxacillin for colony isolation. If only the tube with the PRMB culture changed the colour, 1 ml-aliquot was briefly centrifuged, and the pellet was tested for clumping factor, protein A and staphylococcal polysaccharides (Staphytect plus test, Oxoid) in order to confirm presumptive identification as *S. aureus*. The tubes that did not change colour after 48 h of incubation at 37 °C were considered negative for the presence of both *S. aureus* and MRSA. MRSA-negative samples were subjected to a second screening procedure (look-back) to exclude the presence of MRSA in the first enrichment medium (MHB + 6.5% NaCl) (Additional file 2: Figure S2).

### Molecular typing

Genomic DNA of the MRSA isolates was extracted by QIAamp DNA Mini Kit (QIAGEN) according to the manufacturer’s recommendations, except for the addition of lysostaphin (Sigma Aldridch) at 50 μg/ml for the lysis step. *S. aureus* species identification and methicillin resistance were confirmed as previously described [65] by a multiplex PCR amplifying the genes 16S rDNA, *nuc* and *mecA*. The presence of *pvl* genes (*lukS*-*lukF*) coding for the Panton-Valentine leukocidin (PVL) was tested as previously described [66].

The methods used for the genotyping the MRSA isolates were *spa* typing, MLST and SCC*mec* typing. The PCR to determine the *spa* type was performed as previously described [67]. Briefly, the polymorphic region of *spa* gene was amplified by PCR and the product was double-strand sequenced. The sequences (forward and reverse) were paired and analyzed with the *spa* typing plugin of the BioNumerics software version 6.6 (Applied Maths).

The ST398-specific PCR was carried out with primer sets A07f/A07r [30]. Analysis of the A07 fragment was performed by sequencing the amplicon with primers A07f/A07r.

MLST was performed using the method described by Enright et al. [68]. Seven housekeeping genes (*arcC, aroE, glpF, gmk, pta, tpi, yqiL*) were amplified and sequenced on both DNA strands. The allelic profile and the ST were determined upon interrogation of the *S. aureus* MLST database of (http://saureus.mlst.net).

The SCC*mec* type was determined by a combination of multiplex PCR assays according to a previously described procedure [69, 70]. The multiplex PCRs allow to discriminate the SCC*mec* element based on the amplicon size. Subtypes of the SCC*mec* type IV were determined as described by Milheirico et al. [71].

### Antimicrobial susceptibility testing

Antimicrobial susceptibility was performed by Vitek2 system (bioMérieux), using the AST-P588 card for all strains. Strains were tested for susceptibility to β-lactams (PEN, OXA, AMS); carbapenems (imipenem; IMP); aminoglycosides (GEN, KAN); fluoroquinolones (ENR, MAR); macrolides (ERY); ansamycins (RIF); folate pathway inhibitors (SXT), fucidanes (FUS); lincosamides (CLI); glycopeptides (VAN); tetracyclines (TET); nitrofuranes (nitrofurantoin; NIT); phenicols (CHL). According to the CLSI interpretative criteria [72], MRSA isolates were classified as susceptible, intermediate, or resistant to each antibiotic. Strains classified as resistant and intermediate were included in the same group (non-susceptible).

### Statistical analysis

Data analyses were performed using Sigma Plot software version 12.0 (Systat Software). Categorical variables were compared with the χ^2^ test or Fisher’s exact test when appropriate. *P* values of ≤0.05 were considered statistically significant. Minimum-spanning-tree analysis of *spa* types was performed by using the BioNumerics software version 6.6 (Applied Maths).

## Additional files

Additional file 1: **Figure S1** Geographic distribution of selected pig farms in the Calabria region, Southern Italy. (PDF 285 kb)

Additional file 2: **Figure S2** Flow-chart of *S. aureus* and MRSA screening procedure. (PDF 663 kb)

Additional file 3: **Table S1** Distribution of SCC*mec* types according to the *spa* type. (PDF 12 kb)

Additional file 4: **Figure S3** Sequence of the 1535-nt DNA amplicon containing the IS*256* insertion in the A07 fragment of the SAPIG2195 coding region. (PDF 432 kb)

Additional file 5: **Table S2** Antimicrobial resistance profile and epidemiological type of 219 MRSA isolates. (PDF 34 kb)

Additional file 6: **Table S3** Diversity of antimicrobial susceptibility patterns among MRSA epidemiological types. (PDF 101 kb)

## Abbreviations

AMS: Ampicillin/sulbactam
CA-MRSA: Community-associated methicillin-resistant *Staphylococcus aureus*
CC: Clonal complex
CHL: Chloramphenicol
CS: Cosenza
CZ: Catanzaro
DR: Direct repeat
ECDC: European Centre for Disease Prevention and Control
EMA: European Medicines Agency
ENR: Enrofloxacin
ERY: Erythromycin
ESVAC: European Surveillance of Veterinary Antimicrobial Consumption
EU: European Union
FUS: Fusidic acid
GEN: Gentamicin
HA-MRSA: Healthcare-associated methicillin-resistant *Staphylococcus aureus*
IMP: Imipenem
IR: Inverted repeat
KAN: Kanamycin
KR: Crotone
LA-MRSA: Livestock-associated methicillin-resistant *Staphylococcus aureus*
MAR: Marbofloxacin
MDR: Multi-drug resistant
MHA: Muller Hinton Agar
MLST: Multi Locus Sequence Typing
MRSA: Methicillin-resistant *Staphylococcus aureus*
NCR: Non-coding region
NIT: Nitrofurantoin
OXA: Oxacillin
PDO: Protected Designation of Origin
PEN: Penicillin
PRMB: Phenol-Red Mannitol Broth
PVL: Panton-Valentine leucocidin
RC: Reggio Calabria
RIF: Rifampicin
SCC*mec*: Staphylococcal chromosomal cassette *mec*
ST: Sequence type
SXT: Trimethoprim-sulfamethoxazole
TET: Tetracycline
VAN: Vancomycin
VV: Vibo Valentia
