# Supplementary material for: Prevalence, molecular epidemiology, and antimicrobial resistance of methicillin-resistant Staphylococcus aureus from swine in southern Italy
Source: BMC Microbiol. 2019 Feb 26;19:51. doi: 10.1186/s12866-019-1422-x (PMC6390553; doi:10.1186/s12866-019-1422-x)
Supplement: Supplementary file 2 — Figure S2 Flow-chart of S. aureus and MRSA screening procedure. (PDF 663 kb) [file 12866_2019_1422_MOESM2_ESM.pdf]

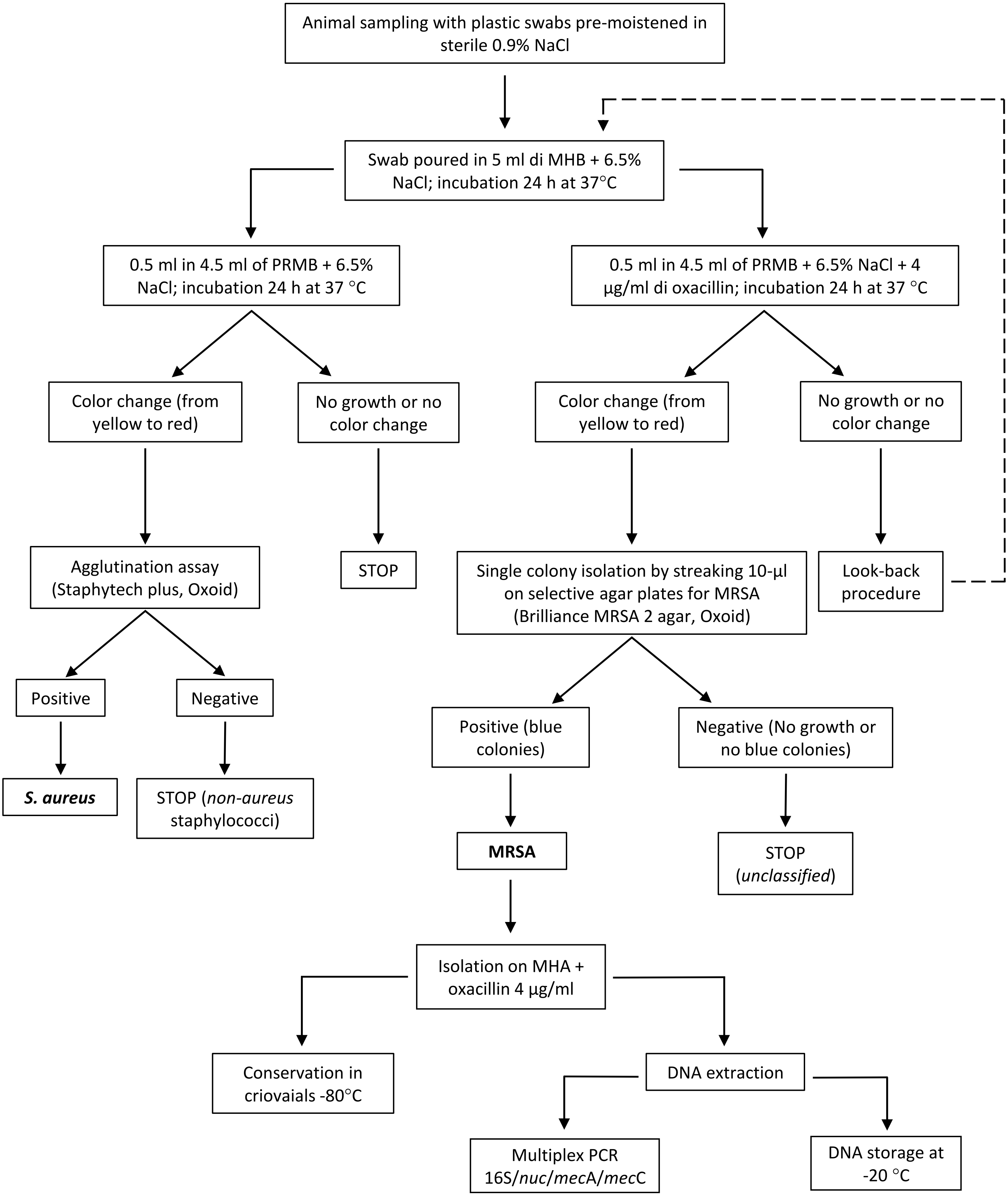

Figure S2. Flow-chart of *S. aureus* and MRSA screening procedure. Abbreviations: MHB, Mueller-Hinton Broth; MHA, Mueller-Hinton Agar; PRMB, Phenol Red Mannitol Broth.
