# Supplementary material for: Prevalence, molecular epidemiology, and antimicrobial resistance of methicillin-resistant Staphylococcus aureus from swine in southern Italy
Source: BMC Microbiol. 2019 Feb 26;19:51. doi: 10.1186/s12866-019-1422-x (PMC6390553; doi:10.1186/s12866-019-1422-x)
Supplement: Supplementary file 3 — Table S1 Distribution of SCCmec types according to the spa type. (PDF 12 kb) [file 12866_2019_1422_MOESM3_ESM.pdf]

Table S1. Distribution of SCC*mec* types according to the *spa* type

***spa* types**	**Repeat succession**	**No. of isolates (%)**	**SCC*mec* type (no., %)**
t011	08-16-02-25-34-24-25	81 (37.0)	V (81, 100%)
t034	08-16-02-25-02-25-34-24-25	49 (22.4)	V (49, 100%)
t899	07-16-23-02-34	33 (15.1)	IVc (11, 33.3%), V (22, 66.7%)
t571	08-16-02-25-02-25-34-25	17 (7.8)	V (17, 100%)
t1606	08-16-34-34-24-25	13 (5.9)	V (13, 100%)
t4474	26-16-23-02-34	12 (5.5)	V (12, 100%)
t10485	35-25-02-25-34-24-25	4 (1.8)	V (4, 100%)
t108	08-16-02-25-24-25	3 (1.4)	V (3, 100%)
t1184	08-16-02-25-25	2 (0.9)	V (2, 100%)
t1793	08-16-02-25-02-25-34-24-24-25	2 (0.9)	V (2, 100%)
t18290	08-16-783-25-34-24-25	1 (0.5)	V (1, 100%)
t2876	08-16-02-25-02-25-24-25	1 (0.5)	V (1, 100%)
t5524	08-16-34-34-25	1 (0.5)	V (1, 100%)
Total		219 (100)	IVc (11, 5.0%), V (208, 95.0%)
