# Supplementary material for: Prevalence, molecular epidemiology, and antimicrobial resistance of methicillin-resistant Staphylococcus aureus from swine in southern Italy
Source: BMC Microbiol. 2019 Feb 26;19:51. doi: 10.1186/s12866-019-1422-x (PMC6390553; doi:10.1186/s12866-019-1422-x)
Supplement: Supplementary file 4 — Figure S3 Sequence of the 1535-nt DNA amplicon containing the IS256 insertion in the A07 fragment of the SAPIG2195 coding region. (PDF 432 kb) [file 12866_2019_1422_MOESM4_ESM.pdf]

tgaccgtaatcttgtaatatttaaaggctaaatttctaatcattttattttgttcagcatcatttaa
ttttctaaaaaaacgcacta**tattttct***GATAAAGTCCGTATAATTGTGTAAAA*GTAAAAAGGCCATA
TAACAGTCCTTTTACGGTACAATGTTTTTAACGACAAAAACATACCCAGGAGGACTTTTACATGACCC
AAGTACATTTTACACTGAAAAGCGAAGAGATTCAAAGCATTATTGAATATTCTGTAAAGGATGACGTT
TCTAAAAATATTTTAACAACGGTATTTAATCAACTAATGGAAAATCAACGAACAGAATATATTCAAGC
AAAAGAATATGAACGAACAGAAAACCGACAAAGTCAACGAAATGGCTATTATGAGCGCAGCTTTACGA
CACGTGTAGGCACGCTAGAATTAAAAGTACCCAGAACACGTGATGGCCATTTTTCACCCACAGTGTTT
GAACGTTATCAACGAAACGAAAAAGCCCTCATGGCTTCAATGTTGGAAATGTATGTATCAGGCGTTTC
AACTCGTAAAGTATCAAAAATTGTGGAAGAACTTTGTGGTAAATCCGTCTCTAAGTCCTTCGTTTCTA
GCTTAACAGAACAGCTAGAAACCTATGGTTAACGAGTGGCAGAATCGTTTATTATCAGAAAAAAATTT
ATTCTTTACTTAATGACCGATGTACTCTATATAAAAGTACGAGAAGAAAATCGAGTACTCTCAAAAAG
CTGTCATATAGCGATTGGAATAACCAAAGATGGCGACCGTGAAATTATCGGCTTCATGATTCAAAGTG
GCGAAAGCGAAGAGACCTGGACAACATTTTTTTGAATACCTAAAAGAACGCGGTTTACAAGGTACGGA
ACTCGTTATTTCTGATGCGCACAAAGGATTAGTCTCTGCCATTAGAAAATCCTTCACCAACGTAAGGT
TGGCAAAGATGCCAAGTTCACTTCCTAAGAAATATCTTTACCACCATTCCTAAAAAAAATTCAAAATC
TTTCAGAGAAGCTGTTAAAGGAATTTTTAAGTTCACAGATATTAACTTAGCGCGTGAGGCTAAAAATC
GATTGATTCATGATTATATCGATCAACCAAAATATTCAAAAGCTTGCGCATCATTGGATGATGGATTC
GAAGACGCCTTTCAATATACCGTACAAGGAAATTCCCACAATCGACTAAAGAGTACCAATCTAATTGA
ACGACTGAATCAAGAAGTACGCAGAAGAGAAAAGATTATTCGCATCTTCCCCAATCAAACATCAGCCA
ATCGCTTAATTGGAGCCGTTCTTATGGACCTACATGATGAATGGATTTATTCTTCAAGAAAATACATC
AATTTTGATAAGTAGAAATGGTAAAAACATTGTATAGCA*TTTTACACAGGAGTCTGGACTTGACT***tat**
tttctgtatcaaatttttttaattccgttagaatttctagttttcttctgtggttaatatctttctta
tgataagctttcatataaatatttaagtattctgggatc

Figure S3. Sequence of the 1,535-nt DNA amplicon containing the IS*256* insertion in the A07 fragment of the SAPIG2195 coding region (lower case). A07r and A07f (reverse-complementary) primer sequences are underlined. The two octanucleotide direct repeats flanking IS*256* are in bold, and the two imperfect inverted repeats flanking the transposase gene are italicized. The coding region of the transposase gene is highlighted in gray.
