# Supplementary material for: Prevalence, molecular epidemiology, and antimicrobial resistance of methicillin-resistant Staphylococcus aureus from swine in southern Italy
Source: BMC Microbiol. 2019 Feb 26;19:51. doi: 10.1186/s12866-019-1422-x (PMC6390553; doi:10.1186/s12866-019-1422-x)
Supplement: Supplementary file 5 — Table S2 Antimicrobial resistance profile and epidemiological type of 219 MRSA isolates. (PDF 34 kb) [file 12866_2019_1422_MOESM5_ESM.pdf]

Table S2. Antimicrobial resistance profile and epidemiological type of 219 MRSA isolates

Antimicrobial resistance profile	N° of isolates (%)	Epidemiological type (*spa* and SCC*mec* type) (no. of isolates)
PEN,AMS,OXA,ENR,MAR,ERY,CLI,TET,SXT	22 (10.0)	t011-V (4/81), t034-V (11/49), t571-V (1/17), t10485-V (2/4), t108-V (2/3), t1793-V (2/2)
PEN,AMS,OXA,CLI,TET	18 (8.2)	t011-V (18/81)
PEN,OXA,CLI,TET,SXT	15 (6.8)	t899-V (10/22), t571-V (1/17), t4474-V (4/12)
PEN,OXA,ENR,MAR,CLI,TET,SXT	13 (5.9)	t034-V (9/49), t4474-V (4/12)
PEN,OXA,ENR,MAR,ERY,CLI,TET,SXT	8 (3.7)	t034-V (7/49), t571-V (1/17)
PEN,AMS,OXA,ENR,MAR,CLI,TET,SXT	7 (3.2)	t034-V (5), t571-V (2/17)
PEN,AMS,OXA,ERY,CLI,TET,SXT	7 (3.2)	t011-V (3/81), t034-V (2/49), t10485-V (1/4), t2876-V (1/1)
PEN,AMS,OXA,CLI,TET,SXT	7 (3.2)	t034-V (2/49), t571-V (5/17)
PEN,AMS,OXA,ENR,CLI,TET	6 (2.7)	t011-V (6/81)
PEN,AMS,OXA,ENR,CLI,TET,SXT	5 (2.3)	t034-V (3/49), t571-V (2/17)
PEN,AMS,OXA,ENR,MAR,ERY,CLI,TET	5 (2.3)	t011-V (5/81)
PEN,AMS,OXA,ENR,ERY,CLI,TET,SXT	5 (2.3)	t011-V (1/81), t034-V (3/49), t10485-V (1/4)
PEN,OXA,ENR,CLI,TET,SXT	5 (2.3)	t899-V (1/22), t571-V (1/17), t4474-V (3/12)
PEN,OXA,GEN,KAN,ENR,MAR,CLI,TET	5 (2.3)	t899-V (5/22)
PEN,OXA,GEN,KAN,ENR,ERY,CLI,TET,SXT	4 (1.8)	t1606-V (4/13)
PEN,OXA,CLI,TET	4 (1.8)	t011-V (4/81)
PEN,AMS,OXA,ENR,MAR,CLI,TET,NIT,SXT	4 (1.8)	t571-V (4/17)
PEN,AMS,OXA,GEN,KAN,ENR,MAR,ERY,TET,SXT	3 (1.4)	t011-V (3/81)
PEN,AMS,OXA,IMP,GEN,KAN,ENR,MAR,ERY,CLI,TET	3 (1.4)	t011-V (3/81)
PEN,OXA,GEN,KAN,ENR,MAR,ERY,CLI,TET,SXT	3 (1.4)	t1606-V (3/13)
PEN,AMS,OXA,KAN,ENR,ERY,CLI,TET,SXT	3 (1.4)	t011-V (1/81), t1184-V (2/2)
PEN,AMS,OXA,GEN,KAN,ENR,TET,SXT	2 (0.9)	t011-V (2/81)
PEN,AMS,OXA,ENR,MAR,ERY,CLI,TET,NIT,SXT	2 (0.9)	t034-V (1/49), t108-V (1/3)
PEN,AMS,OXA,IMP,ENR,MAR,ERY,CLI,TET,SXT	2 (0.9)	t011-V (1/81), t034-V (1/49)
PEN,AMS,OXA,IMP,GEN,KAN,ERY,CLI,TET,SXT	2 (0.9)	t1606-V (1/13), t5524-V (1/1)
PEN,AMS,OXA,GEN,KAN,CLI,TET	2 (0.9)	t011-V (2/81)
PEN,AMS,OXA,IMP,ENR,ERY,CLI,TET,SXT	2 (0.9)	t011-V (1/81), t034-V (1/49)
PEN,AMS,OXA,ERY,CLI,TET	2 (0.9)	t011-V (2/81)
PEN,OXA,ENR,MAR,CLI,TET,CHL,SXT	2 (0.9)	t899-IVc (2/11)
PEN,AMS,OXA,ENR,MAR,ERY,CLI,TET,CHL,SXT	2 (0.9)	t899-IVc (2/11)
PEN,OXA,CLI,TET,CHL,SXT	2 (0.9)	t899-IVc (2/11)
PEN,OXA,GEN,KAN,ENR,MAR,TET	2 (0.9)	t899-V (2/22)
PEN,AMS,OXA,GEN,KAN,ENR,MAR,CLI,TET,SXT	1 (0.5)	t011-V (1/81)
PEN,AMS,OXA,IMP,GEN,KAN,ENR,MAR,TET,SXT	1 (0.5)	t011-V (1/81)
PEN,AMS,OXA,GEN,KAN,ENR,TET	1 (0.5)	t011-V (1/81)
PEN,AMS,OXA,GEN,KAN,TET,SXT	1 (0.5)	t011-V (1/81)
PEN,OXA,KAN,ENR,MAR,ERY,CLI,TET,CHL,SXT	1 (0.5)	t899-IVc (1/11)
PEN,AMS,OXA,IMP,KAN,ENR,MAR,ERY,CLI,TET,CHL,SXT	1 (0.5)	t011-V (1/81)
PEN,AMS,OXA,IMP,ENR,MAR,ERY,CLI,TET,CHL,SXT	1 (0.5)	t011-V (1/81)
PEN,AMS,OXA,ENR,MAR,TET,CHL	1 (0.5)	t011-V (1/81)
PEN,OXA,ENR,MAR,ERY,CLI,TET,CHL	1 (0.5)	t011-V (1/81)
PEN,OXA,GEN,KAN,ERY,CLI,TET,SXT	1 (0.5)	t1606-V (1/13)
PEN,AMS,OXA,GEN,KAN,ENR,ERY,CLI,TET,SXT	1 (0.5)	t1606-V (1/13)
PEN,AMS,OXA,IMP,GEN,KAN,CLI,TET	1 (0.5)	t011-V (1/81)
PEN,AMS,OXA,GEN,KAN,ENR,MAR,CLI,TET	1 (0.5)	t011-V (1/81)
PEN,AMS,OXA,GEN,KAN,ENR,MAR,ERY,CLI,TET	1 (0.5)	t011-V (1/81)
PEN,AMS,OXA,GEN,KAN,ENR,MAR,ERY,CLI,TET,NIT,SXT	1 (0.5)	t011-V (1/81)
PEN,AMS,OXA,IMP,GEN,KAN,ENR,MAR,ERY,CLI,TET,SXT	1 (0.5)	t1606-V (1/13)
PEN,AMS,OXA,GEN,KAN,ERY,CLI,TET	1 (0.5)	t011-V (1/81)
PEN,AMS,OXA,IMP,ENR,MAR,ERY,TET,SXT	1 (0.5)	t1606-V (1/13)
PEN,AMS,OXA,IMP,GEN,KAN,ENR,ERY,CLI,TET,SXT	1 (0.5)	t1606-V (1/13)
PEN,AMS,OXA,IMP,GEN,KAN,ERY,CLI,TET	1 (0.5)	t18290-V (1/1)
PEN,AMS,OXA,GEN,ENR,MAR,ERY,CLI,TET	1 (0.5)	t011-V (1/81)
PEN,AMS,OXA,ENR,MAR,ERY,CLI,TET,RIF,SXT	1 (0.5)	t011-V (1/81)
PEN,AMS,OXA,IMP,ENR,ERY,CLI,TET	1 (0.5)	t011-V (1/81)
PEN,OXA,MAR,CLI,TET	1 (0.5)	t011-V (1/81)
PEN,AMS,OXA,MAR,ERY,CLI,TET,CHL,SXT	1 (0.5)	t011-V (1/81)
PEN,AMS,OXA,IMP,ERY,CLI,TET	1 (0.5)	t011-V (1/81)
PEN,AMS,OXA,IMP,CLI,TET	1 (0.5)	t011-V (1/81)
PEN,AMS,OXA,IMP,KAN,ENR,MAR,ERY,CLI,TET	1 (0.5)	t034-V (1/49)
PEN,AMS,OXA,IMP,CLI,TET,SXT	1 (0.5)	t034-V (1/49)
PEN,AMS,OXA,KAN,ENR,MAR,ERY,CLI,TET,CHL,SXT	1 (0.5)	t899-IVc (1/11)
PEN,AMS,OXA,ERY,CLI,TET,CHL,SXT	1 (0.5)	t899-IVc (1/11)
PEN,OXA,ERY,CLI,TET,CHL,SXT	1 (0.5)	t899-IVc (1/11)
PEN,OXA,ENR,MAR,ERY,CLI,TET,CHL,SXT	1 (0.5)	t899-IVc (1/11)
PEN,AMS,OXA,ENR,CLI,TET,NIT,SXT	1 (0.5)	t034-V (1/49)
PEN,AMS,OXA,CLI,TET,NIT,SXT	1 (0.5)	t034-V (1/49)
PEN,AMS,OXA,ENR,CLI,TET,NIT	1 (0.5)	t011-V (1/81)
PEN,AMS,OXA,GEN,CLI,TET	1 (0.5)	t011-V (1/81)
PEN,AMS,OXA,GEN,ERY,CLI,TET,SXT	1 (0.5)	t011-V (1/81)
PEN,OXA,ENR,ERY,CLI,TET	1 (0.5)	t899-V (1/22)
PEN,OXA,TET	1 (0.5)	t899-V (1/22)
PEN,OXA,ENR,MAR,ERY,CLI,TET	1 (0.5)	t899-V (1/22)
PEN,OXA,ENR,TET	1 (0.5)	t899-V (1/22)
PEN,OXA,MAR,CLI,TET,SXT	1 (0.5)	t4474-V (1/12)
PEN,AMS,OXA,ENR,MAR,CLI,TET	1 (0.5)	t011-V (1/81)
PEN,AMS,OXA,ENR,MAR,TET	1 (0.5)	t011-V (1/81)
