# Supplementary material for: Prevalence, molecular epidemiology, and antimicrobial resistance of methicillin-resistant Staphylococcus aureus from swine in southern Italy
Source: BMC Microbiol. 2019 Feb 26;19:51. doi: 10.1186/s12866-019-1422-x (PMC6390553; doi:10.1186/s12866-019-1422-x)
Supplement: Supplementary file 6 — Table S3 Diversity of antimicrobial susceptibility patterns among MRSA epidemiological types. (PDF 101 kb) [file 12866_2019_1422_MOESM6_ESM.pdf]

Table S3. Diversity of antimicrobial susceptibility patterns among MRSA epidemiological types

Epidemiological type (*spa* and SCC*mec* type)	No. of isolates	No. of antibiotypes	Antibiotype diversity[a]
t011-V	81	40	0.51
t034-V	49	15	0.69
t899-V	22	8	0.64
t899-IVc	11	8	0.27
t571-V	17	8	0.53
t1606-V	13	8	0.38
t4474-V	12	4	0.67
t10485-V	4	3	0.25
t108-V	3	2	0.33
t1184-V	2	1	0.50
t1793-V	2	1	0.50
t18290-V	1	1	0
t2876-V	1	1	0
t5524-V	1	1	0
Total	219	101	0.54

[a] Expressed as 1 – (No. of antibiotypes / No. of isolates)
