# Supplementary material for: Prevalence, molecular epidemiology, and antimicrobial resistance of methicillin-resistant Staphylococcus aureus from swine in southern Italy
Source: BMC Microbiol. 2019 Feb 26;19:51. doi: 10.1186/s12866-019-1422-x (PMC6390553; doi:10.1186/s12866-019-1422-x)
Supplement: Supplementary file 1 — Figure S1 Geographic distribution of selected pig farms in the Calabria region, Southern Italy. (PDF 285 kb) [file 12866_2019_1422_MOESM1_ESM.pdf]

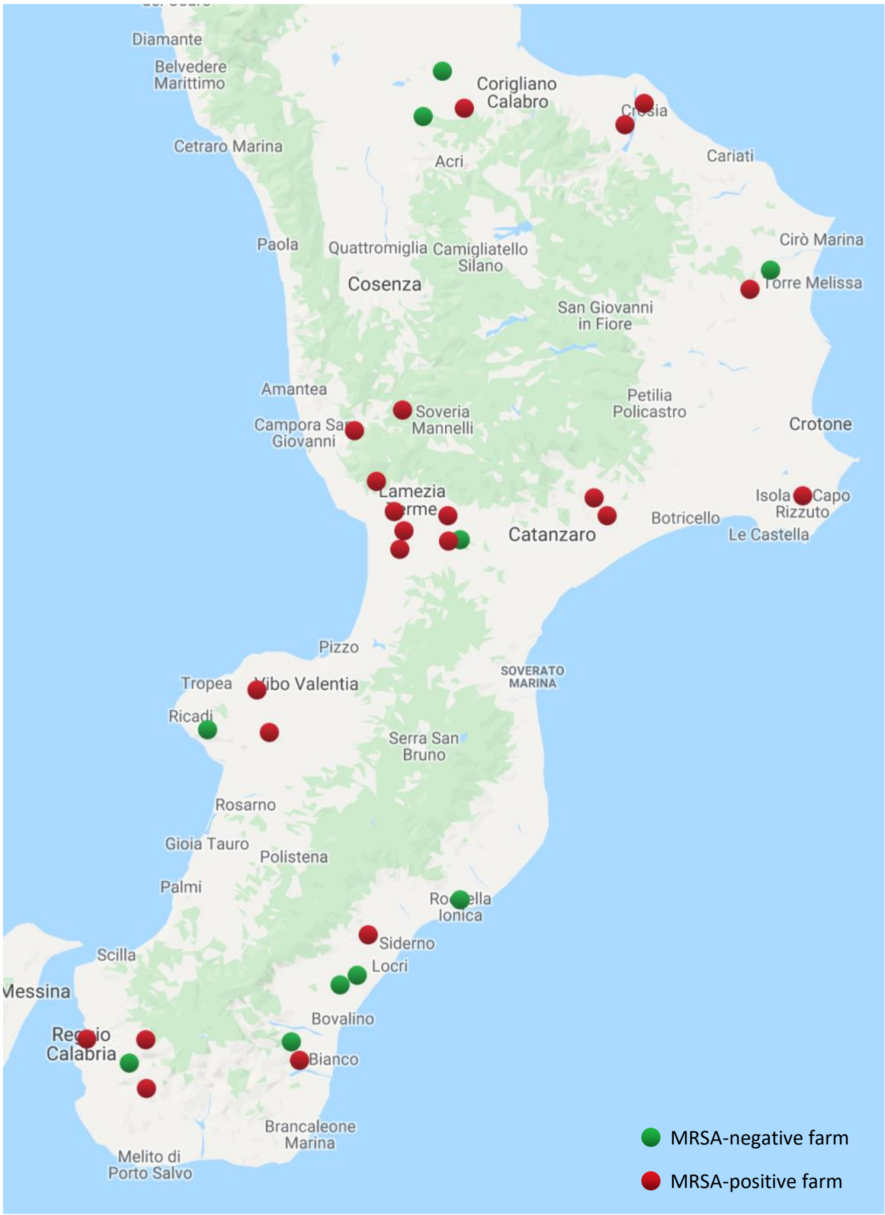

Figure S1. Geographic distribution of selected pig farms in the Calabria region, Southern Italy. Red and green dots denote MRSA-positive and -negative farms, respectively.
